# A Feasibility Study on 3D Bioprinting of Microfat Constructs Towards Wound Healing Applications

**DOI:** 10.3389/fbioe.2021.707098

**Published:** 2021-07-27

**Authors:** Trevor Schmitt, Nathan Katz, Vipuil Kishore

**Affiliations:** ^1^Department of Biomedical and Chemical Engineering and Sciences, Florida Institute of Technology, Melbourne, FL, United States; ^2^Jointechlabs Inc., North Barrington, IL, United States

**Keywords:** 3D printing, microfat, collagen, chronic wounds, wound healing

## Abstract

Chronic wounds affect over 400,000 people in the United States alone, with up to 60,000 deaths each year from non-healing ulcerations. Tissue grafting (e.g., autografts, allografts, and xenografts) and synthetic skin substitutes are common treatment methods, but most solutions are limited to symptomatic treatment and do not address the underlying causes of the chronic wound. Use of fat grafts for wound healing applications has demonstrated promise but these grafts suffer from low cell viability and poor retention at the wound site resulting in suboptimal healing of chronic wounds. Herein, we report on an innovative closed-loop fat processing system (MiniTC^TM^) that can efficiently process lipoaspirates into microfat clusters comprising of highly viable regenerative cell population (i.e., adipose stromal cells, endothelial progenitors) preserved in their native niche. Cryopreservation of MiniTC^TM^ isolated microfat retained cell count and viability. To improve microfat retention and engraftment at the wound site, microfat was mixed with methacrylated collagen (CMA) bioink and 3D printed to generate microfat-laden collagen constructs. Modulating the concentration of microfat in CMA constructs had no effect on print fidelity or stability of the printed constructs. Results from the Alamar blue assay showed that the cells remain viable and metabolically active in microfat-laden collagen constructs for up to 10 days *in vitro*. Further, quantitative assessment of cell culture medium over time using ELISA revealed a temporal expression of proinflammatory and anti-inflammatory cytokines indicative of wound healing microenvironment progression. Together, these results demonstrate that 3D bioprinting of microfat-laden collagen constructs is a promising approach to generate viable microfat grafts for potential use in treatment of non-healing chronic wounds.

## Introduction

Chronic skin wounds, such as diabetic foot ulcers and pressure ulcers, are highly prevalent in patients with poor circulation, neuropathy, and infection (Gefen, 2019). Current treatment strategies primarily entail tissue debridement to remove necrotic tissue, off-loading to alleviate pressure on the ulcer site, and application of analgesics or antibiotics to reduce pain and prevent infection. Despite these interventions, 14–24% of diabetic patients with foot ulcers undergo amputation and are associated with mortality of up to 80% at 5 years post amputation (Jupiter et al., 2015; Thorud et al., 2016; Tresierra-Ayala and García Rojas, 2017). In a similar vein, approximately 60,000 deaths occur each year due to complications related to non-healing pressure ulcers (Berlowitz et al., 2012). Tissue grafting through autografts, allografts, or xenografts are routinely used in the clinic for the treatment of chronic skin wounds, but limitations such as availability, immune rejection, and possible disease transmission are major concerns (Casadei et al., 2012; Wang et al., 2013). To circumvent these complications, tissue-engineered skin substitutes (e.g., Hyalograft 3D, Dermagraft^®^) are currently available in the market to promote regeneration of skin lesions and ulcerations. However, there are several unresolved limitations with the use of these commercial products that include poor vascularization, fibrous capsule formation, and immune complications (Vig et al., 2017). Therefore, there is an unmet need for a viable full-thickness skin substitute that employs autologous cells and promotes neovascularization for effectual and complete treatment of chronic skin wounds.

Adipose tissue represents an attractive source of autologous cells for regenerative therapy due to its abundance and surgical accessibility. Application of adipose-derived cells has been shown to be a promising approach toward the treatment of non-healing wounds (van Dongen et al., 2018). The regenerative cell population in adipose tissue includes adult stem cells, angiogenic progenitors, pericytes, vascular smooth muscle cells, and different types of immune cells. All together these cells called the stromal vascular fraction (SVF) present a powerful paracrine machinery capable of angiogenic, immunomodulatory, and inflammatory regulatory effects (Gimble et al., 2011; Dai et al., 2016). Previous work has shown that SVF incorporation promotes vascularization in tissue constructs via secretion of proangiogenic growth factors (i.e., VEGF, bFGF; Sheng et al., 2011; Zhan et al., 2016). Application of SVF has also been shown to improve burn wound healing in rats via increased cell proliferation, well-regulated inflammation, and improved vascularization (Atalay et al., 2014). Despite these promising outcomes, poor cell retention rate and limited engraftment and survival at the wound site are major concerns. Micronized fat tissue (i.e., microfat) obtained from mechanical processing of lipoaspirate preserves the SVF in its native niche and can therefore provide higher cellular yield, greater viability, and improved vascularization, advancing toward the development of more effective therapies for the treatment of chronic wounds (Alharbi et al., 2013; Bianchi et al., 2013). In this realm, Jointechlabs has developed a novel fat processing device (MiniTC^TM^) that allows for closed loop bedside processing of small samples of autologous fat into 3D distinct clusters called microfat. This method preserves the microvessels and associated SVF within the natural niche and eliminates unwanted debris, red blood cells, anesthetic and oil residuals following lipoaspiration. Application of the MiniTC^TM^ technology for instant and highly efficient processing of small samples of adipose tissue can be highly meritorious and aid in the production of a clinically relevant cell enriched autologous microfat grafts for use in wound healing applications.

3D bioprinting is a rapidly evolving field that allows for the generation of cell-laden custom-designed complex tissue constructs with precise ratios of hydrogel carrier and biological material for tissue engineering applications (Cornelissen et al., 2017; Gopinathan and Noh, 2018). Microfat incorporation into polymeric bioinks will allow crosslinking of the construct post printing and thereby enable the generation of mechanically stable 3D structures with controlled depth for use in grafting of deep chronic wounds. Furthermore, application of mechanically stable microfat-laden polymeric grafts can significantly improve engraftment and survival of the fat tissue at the wound site. Due to these advantages, combinatorial printing of microfat (with cells preserved in their native niche) and biocompatible polymers can be a viable approach to deliver 3D fat grafts rich in both adipose stromal cells (ASCs) and endothelial progenitor cells (EPCs) for successful engraftment, enhanced angiogenesis, and expedited healing of chronic skin wounds.

The goals of this study were to: (a) characterize cell viability and cell population in microfat obtained via lipoaspirate processing using an innovative closed loop lipoaspirate processing device (MiniTC^TM^), and (b) demonstrate feasibility to 3D bioprint microfat-laden collagen grafts for potential use in wound healing applications. Mini-TC^TM^ (Jointechlabs, N. Barrington, IL, United States) is equipped with a sterile fluid/tissue pathway for production of micronized fat fraction from patient-derived lipoaspirates. Cell count, viability, and cell subpopulations in freshly isolated and cryopreserved microfat were characterized using Guava Muse Cell Analyzer (Luminex) and flow cytometry. Further, varying ratios of microfat were mixed with methacrylated collagen (CMA) bioink and the feasibility to 3D print viable microfat-laden collagen grafts was investigated by assessing print fidelity and stability, cell metabolic activity, and cytokine secretory activity of the microfat graft.

## Materials and Methods

### Isolation of Microfat From Lipoaspirate Using MiniTC^TM^

**FIGURE 1:**
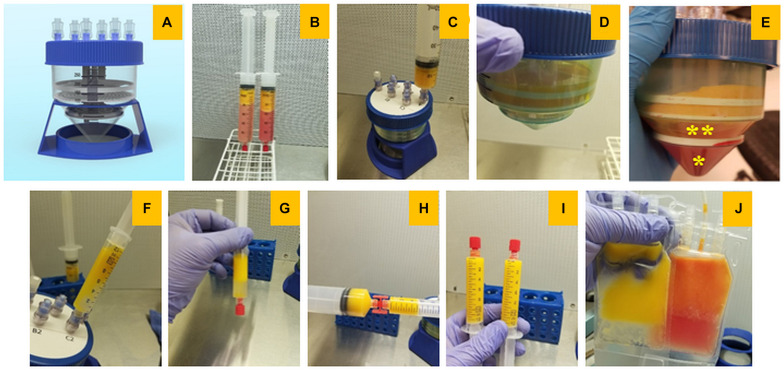
Isolation of microfat from lipoaspirate using MiniTC^TM^ device. **(A)** Image of the MiniTC^TM^ microfat isolation device, **(B)** freshly collected lipoaspirate from adipose tissue, **(C,D)** injection of microfat through the Mini-TC port for centrifugation and processing, **(E)** post-centrifugation microfat clusters accumulate in the intermediate compartment of MiniTCTM (* – red blodd cells and debris, ** – desired fat fraction), **(F)** collection of processed microfat through the luer on top of the device, **(G,H)** separation of excess fluid and homogenization of microfat, **(I)** syringes of microfat ready to use, **(J)** cryopreservation of microfat constituents.

Lipoaspirate was obtained from adipose tissue of living donors with informed consent by following the recommended instructions. Tumescent anesthesia was administered by delivering a large volume of dilute anesthetic solution to subcutaneous adipose tissue prior to a liposuction procedure until the tissue is firm and swollen or truly “tumescent.” Lipoaspirate was obtained following manual lipoaspiration, utilizing 2 mm cannula (Jointechlabs), connected to VacLok^R^ 60 cc syringe (MedEcart). Isolation of microfat from lipoaspirate using the MiniTC^TM^ system involves multiple steps as described in Figure 1. Primarily, MiniTC^TM^ entails two simple centrifugation steps for obtaining injectable microfat in about 20 min. Step one eliminates the blood, debris and anesthetic contaminants, and step two allows for gentle dispersion of dense fat into distinct microfat clusters of range 400–700 μm, where regenerative stromal cells are preserved within their natural niche. MiniTC^TM^ internal design consisted of 3 compartments: upper, middle, and lower. The upper compartment is between the top sealed plate and tissue dispersion screen; the middle is between the screen and the bottom filter located in the conical part of the device, and the lower compartment is below the filter. Firstly, the collected lipoaspirate was decanted and the dense fraction was introduced into the MiniTC^TM^ system through valved inlets, accumulating on the surface of the screen. The fat fraction was washed with 50 ml of Lactated Ringer solution (Baxter) and centrifuged at 250 g for 5 min (Biospin Centrifuge, Jointechlabs) which results in all excess oil, blood, and debris to be washed down and collected in the bottom compartment. Following this, MiniTC^TM^ device was filled with fresh washing solution and spun again at 500 g for 15 min for gentle separation of micronized fat clusters through a separation screen with proprietary configuration and size of pores. Post centrifugation, the microfat accumulated in the intermediate compartment between the screen and the filter was collected by connecting the syringe to the luer on top of the device. The collected microfat clusters was then gently homogenized to obtain fine washed microfat graft and used for all the experiments.

### Cryopreservation and Thawing of Microfat

MiniTC^TM^ prepared microfat was mixed with equal volume of cryopreservation solution (Stem-CellBanker^R^, AMSBIO) in a CF-50 (Origen) cryobag. The mixture was placed into a CF-50 storage cassette, incubated at –20°C for 15 min, and plunged into liquid nitrogen. For 3D printing experiments, a similar freezing protocol was employed but the microfat was a mixed with cryopreservation solution and aliquoted into 2 ml cryovials prior to freezing. Microfat samples were shipped on dry ice to Florida Institute of Technology and returned into liquid nitrogen storage until use. On the day of the experiments, the microfat was thawed by immersing the cryovials in a 37°C water bath. After thawing, the microfat was collected using a blunt 16 g needle into a 5 ml syringe containing 2 ml of Lactated Ringer solution. Keeping the syringe upright, the needle was removed and replaced by a luer cap and the content was mixed well by flipping the syringe upside down several times. Following this, the syringe was left in a vertical position with the luer cap facing downward for 5 min, after which the luer cap was removed and the liquid phase was allowed to spill out. Utilizing a double luer connector (Rapid-Fill, Baxter) the dense micronized fat fraction was transferred into a 1 ml syringe and used for 3D printing experiments.

### Assessment of Cell Count and Viability in SVF of Microfat

Lipoaspirates were collected from three different patients with informed consent and processed using the Mini-TC device to obtain fresh microfat. A portion of the fresh microfat was cryopreserved by following the protocol described in section “Cryopreservation and thawing of microfat.” Samples of fresh microfat and post cryo-thawed cycle were analyzed in terms of stromal cell content for cell count and viability by using a single channel Muse^R^ Cell Analyzer (Luminex). At first, freshly prepared and cryo-thawed microfat was digested using collagenase NB5 using a Mini-Stem System, which is a version of MiniTC^TM^, intended for the harvest of adipose-derived SVF. The enzymatic digestion process entailed exposing the microfat samples to NB5 collagenase (Nordmark-Pharma, Germany) at enzymatic activity of 0.22–0.25 Wunsch per gram of tissue for 40 min at 37°C. Digestion was stopped via addition of cold washing solution and the cells fraction was pelleted by centrifugation at 800 g for 20 min. Cell suspensions were collected into Ringer Lactate solution supplemented with 10% fetal calf serum and assayed using the MuseR Cell Analyzer within 24 h.

### Characterization of Cell Subpopulations in Microfat

Assessment of cell subpopulations in microfat was performed using a multichannel flow cytometry assay (LSR Fortessa SORP flow cytometer, Becton Dickinson). The sample of microfat obtained with MiniTC^TM^ was frozen as described in section “Cryopreservation and thawing of microfat” and thawed after 8 days of storage in liquid nitrogen. After thawing, the microfat was digested using collagenase in the Mini-Stem system to obtain the SVF cell suspension. Samples were prepared for flow cytometry using a simple wash-stain-wash technique. Briefly, 100 μl of cell suspension was added to 5 ml polystyrene tubes (2 tubes for 2 panels and additional 8 tubes for compensation control/unstained control). The cells were then washed twice with 2 ml PBS + 2% bovine serum albumin (BSA) and resuspended in 50 μl of PBS + 2%BSA solution for blocking. Staining was carried out using antibodies at a predetermined concentration (based on antibody titration) and incubating the tubes in dark at 4°C for 45 min (Panels included in Table 1). Following incubation, the cells were washed again and resuspended in 300 μl of PBS and acquired within an hour on the flow cytometer. The Fortessa is a 16-color capable bench-top multi-laser flow cytometer equipped with six lasers. Excitation lines are at 488, 355, 405, 552, 640, and 685 nm wavelengths. Logarithmic amplifier linearity and dynamic range were tested with Rainbow beads (Spherotech, Libertyville, IL, United States). Compensation settings were adjusted using single stained samples. Detailed analysis of the samples was done using FlowJo (Treestar, Inc.).

**TABLE 1:** Parameters for 3D bioprinting of microfat-laden collagen constructs.

Parameter	Value	Description
Tip diameter	0.965 mm	A blunt syringe tip for the print head
Tip gage	18 g	
Print shape	Cube	Outer limit of construct is square
Infill pattern	Mesh	Inner pattern is cross-hatched
Infill angle	45°	The angle at which the bioink was extruded
Flow speed	5 mm/s	Extrusion speed for optimal density
Support bath	FRESH	Thermoreversible support bath for layer-by-layer printing using soft hydrogel-based bioinks
Temperature	RT	FRESH printing was performed at RT followed by incubation at 37°C to recover the printed constructs
Crosslink power	11 mW/cm^2^	Power density of UV crosslinking
Crosslink time	60 s	Time exposure of UV for crosslinking

### 3D Printing of Microfat Laden Collagen Constructs

Microfat was thawed as described in section “Cryopreservation and thawing of microfat” and agitated gently between two syringes to break up agglomerated fat without adversely affecting the cellular content. Following this, microfat was mixed with 6 mg/mL methacrylated collagen solution (CMA; Advanced Biomatrix, Carlsbad, CA, United States) to the desired experimental concentrations (10, 30, and 50% – w/w). Then, 1% w/v 2,2′-Azobis[2-methyl-N-(2-hydroxyethyl)propionamide] (VA-086) photoinitiator (Wako, Japan) was mixed with 8.5% v/v neutralization solution and added to the CMA-microfat mixture and placed in a syringe equipped with a 18G blunt-tipped needle. Printing was performed using a REGEMAT3D (Granada, Spain) modular-head extrusion bioprinter using the print parameters summarized in Table 1. Freeform reversible embedding of suspended hydrogels technique to produce micronized fat laden 3D constructs with good print and shape fidelity (Hinton et al., 2015). Post printing, the constructs were crosslinked by UV exposure (365 nm; 17 mW/cm^2^) for 1 min. High quality digital images were taken at a fixed distance using a DSLR camera for qualitative assessment of fidelity and stability of 3D printed constructs. Further, line measurement tool on Image J was used to measure the line width microfat of printed constructs for quantitative assessment of the effect of microfat incorporation on the print fidelity of collagen constructs.

### Cell Culture and Quantification of Cell Metabolic Activity

3D printed collagen constructs with different concentrations of microfat were cultured individually in a 12 well plate in growth medium composed of DMEM supplemented with 10% FBS and 1% penicillin/streptomycin for 2 weeks. Culture medium was replaced every 3 days. Cell metabolic activity was quantified using the AlamarBlue assay by following the manufacturer’s instructions. Briefly, at periodic intervals, microfat-laden collagen constructs were incubated in 500 μl of 10% alamar blue mix (i.e., DMEM + 10% alamar blue solution) for 12 h. Following this, the alamar blue mix from each well was transferred into a 96-well plate in triplicate and the relative fluorescence units were measured at 555 nm excitation and 595 nm emission wavelengths using an M2e Spectramax plate reader (Molecular Devices).

### Measurement of Key Wound Healing Cytokines Produced by Microfat in Culture

To quantify the soluble inflammatory factors released from microfat-laden collagen constructs during culture, growth medium that was collected on days 3, 6, 9, 12, and 15 and frozen at –80°C. Frozen samples were shipped to Eve Technologies (Alberta, Canada) and a Human Cytokine 48-Plex Discovery Enzyme-Linked Immunosorbent Assay (ELISA) was performed for assessment of relevant inflammatory markers. Six inflammatory markers are reported in this study which include interleukin-6 (IL-6), interleukin-8 (IL-8), fibroblast growth factor-2 (FGF-2), macrophage colony-stimulating factor (M-CSF), monokine induced gamma interferon (MIG), and Regulated on Activation, Normal T Cell Expressed and Secreted (RANTES).

### Statistical Analyses

Results for cell count and viability for freshly isolated and cryopreserved microfat, print fidelity, and Alamar blue assay are reported as mean ± standard deviation for a sample size of *N* = 3/group. ELISA data for expression of micronized fat secreted cytokines is reported as the mean of two data points. Statistical analysis was performed using one-way ANOVA with Tukey *post hoc* comparisons. Significance criterion was set at *p* < 0.05.

## Results

### Effect of Cryopreservation on Cell Count and Viability in MicroFat

Results for MNC count per gram of freshly processed and cryopreserved microfat from three patients ranged from 0.84 × 10^6^ to 1 × 10^6^ and 0.8 × 10^6^ to 1.05 × 10^6^, respectively, with a comparable average of around 0.9 × 10^6^ for both conditions (Table 2). In similar vein, the percent cell viability ranged from 78–93% with an average of 85% and 88% for freshly processed and cryopreserved fat, respectively. These results indicate that cryopreservation had no effect on MNC count and percent cell viability in microfat processed using the Mini-TC device.

**TABLE 2:** Cell count and percent cell viability – fresh vs. cryopreserved microfat.

	Mononuclear cell count/gram of microfat		Percent cell viability	
		
Microfat condition	Fresh	Cryopreserved	Fresh	Cryopreserved
Patient 1	0.84 × 10^6^	0.80 × 10^6^	90%	93%
Patient 2	0.94 × 10^6^	0.92 × 10^6^	78%	82%
Patient 3	1 × 10^6^	1.05 × 10^6^	88%	90%

### Analyses of Cell Subpopulations in Microfat

**FIGURE 2:**
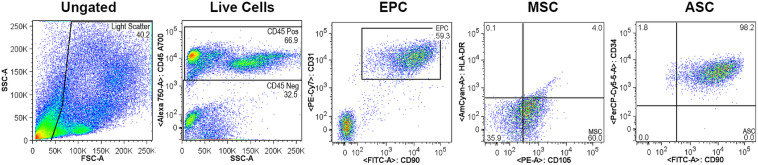
Flow cytometry analyses of cell subpopulations in cryopreserved microfat.

Cell subpopulations in microfat were assessed via flow cytometry post enzymatic digestion of the Mini-TC processed microfat to obtain the cellular fraction. At first, the total harvested live cellular fraction was gated using the CD45 hematopoietic cell marker and the CD45- non-hematopoietic cell population was used for further analyses. Gating results showed that hematopoietic lineage positive for CD45 and non-hematopoietic negative for CD45 demonstrated a split of 67% to 33%, respectively (Figure 2). Of the CD45- cell population, 59% of the cells were double positive for CD90 and CD31, which are known to be expressed by EPCs. Nearly the entire CD45- population expressed CD34 and CD90 cell surface markers (∼98%) which are specific to freshly harvested SVF together with the outer adventitial stromal ring and EPCs in a cell population characterized as ASCs. Further, 60% of the CD45- cells were CD105 + and HLA-DR- which are MSC-like cells and include mostly the adhered population of MSCs and fibroblasts. Together, these results indicate that Mini-TC processed and cryopreserved microfat is a cell enriched fat graft consisting of viable SVF and regenerative cells.

### Effect of Microfat Concentration on Print Fidelity

**FIGURE 3:**
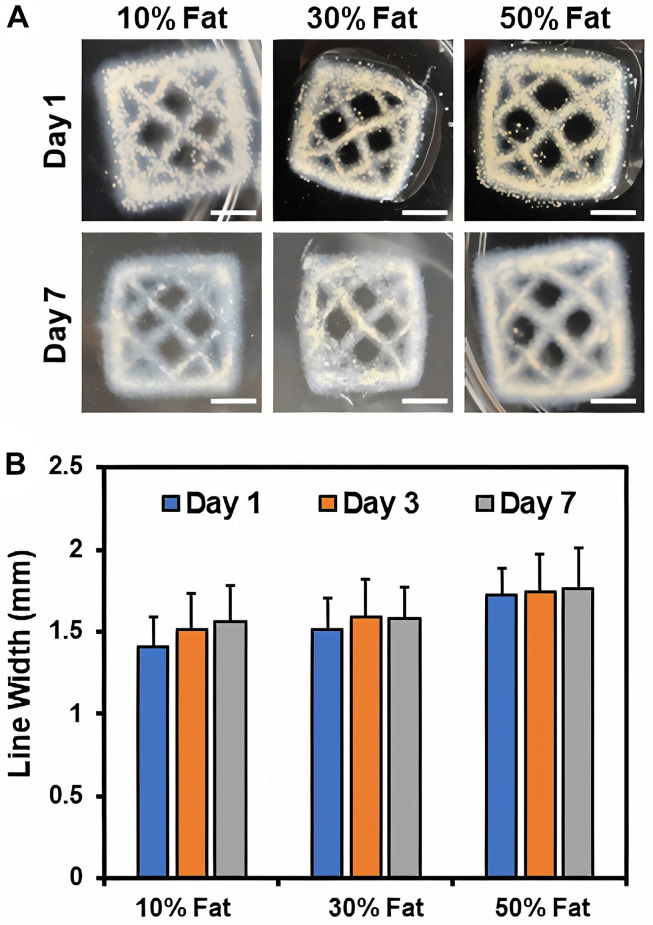
**(A)** High quality images of 3D printed microfat-laden collagen constructs with different concentrations of microfat over time. Scale bar: 5 mm. **(B)** Line width measurement of the inner mesh for assessment of print fidelity and stability of microfat-laden 3D printed collagen constructs.

The effects of microfat incorporation on print fidelity and stability of 3D printed collagen constructs was assessed via visual examination of the constructs over time and quantitative measurements of line width using ImageJ analyses. Results showed that print and shape fidelity of the printed constructs was maintained over time for all microfat compositions indicating that microfat-laden collagen constructs are stable (Figure 3A). Further, microfat appeared to be homogeneously distributed within the 3D bioprinted collagen constructs. Quantitative measurements revealed that the line width of printed constructs was consistent for all microfat compositions indicating that microfat incorporation had no effect on print fidelity of collagen constructs (Figure 3B). Further, no change in construct dimensions was observed over time confirming that microfat-laden collagen constructs are stable. These results indicate that microfat incorporation maintains print fidelity and stability of 3D printed collagen constructs.

### Cell Metabolic Activity in Microfat Laden 3D Printed Constructs

**FIGURE 4:**
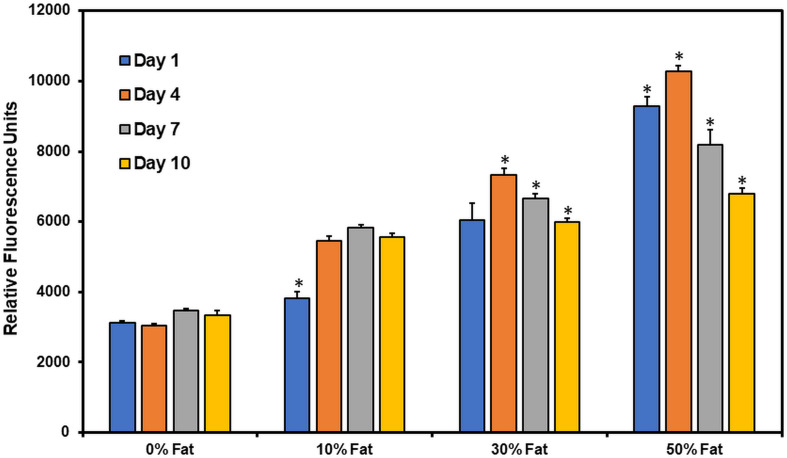
Quantification of cell metabolic activity in microfat-laden collagen constructs over time using Alamar blue assay. (* indicates *p* < 0.05 when comparing between different time points for the same microfat concentration).

Cell metabolic activity in microfat-laden 3D printed collagen constructs was measured using the Alamar blue assay (Figure 4). At day 1, cell metabolic activity of microfat-laden constructs was significantly higher than control constructs (no microfat) in a composition dependent manner indicating that the Alamar blue assay can reliably assess cell metabolic activity in microfat-laden constructs. A significant increase (*p* < 0.05) in cell metabolic activity was observed on all microfat-laden constructs from day 1 to day 4. Cell metabolic activity in 10 and 30% microfat compositions at day 7 was significantly higher than the baseline activity measured at day 1. By day 10, cell metabolic activity was observed to decline for all microfat compositions. Together, these results suggest that cells remain viable and metabolically active in microfat-laden collagen constructs for up to 10 days in *in vitro* culture.

### Temporal Secretion of Wound Healing Cytokines From Microfat in *in vitro* Culture

**FIGURE 5:**
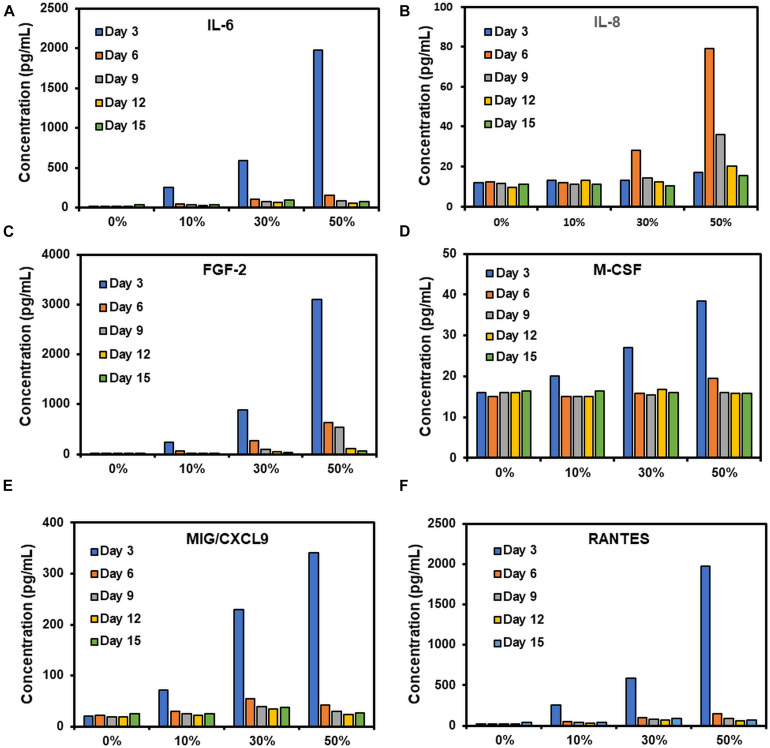
**(A–F)** Assessment of temporal expression of wound healing cytokines from microfat-laden collagen constructs *in vitro*.

An ELISA analysis was performed to quantify the temporal expression of pro-inflammatory cytokines released from microfat-laden constructs in culture. IL-6 expression showed a microfat composition dependent large spike on day 3 for all conditions, followed by a steep decrease in expression by day 6 which continued to stay low for the rest of the culture (Figure 5A). For IL-8, the 0 and 10% microfat conditions showed similar values at all time points indicating that incorporation of low amounts of microfat does not trigger IL-8 release (Figure 5B). On the other hand, IL-8 expression in constructs with higher concentrations of microfat (i.e., 30 and 50%) showed a substantial increase followed by a steady decline for the duration of the culture. The expression profile for FGF-2, MIG/CXCL9, and RANTES was comparable to that of IL-6 for all conditions, showing a microfat concentration dependent spike on day 3 followed by a drop in expression from day 6 through day 15 of the culture although the expression levels were consistently higher than the no microfat control at all time points (Figures 5C,E,F). M-CSF showed a microfat concentration dependent spike on day 3, though quickly leveling back down to values comparable to the control by day 6 (Figure 5D). These results suggest that microfat-laden collagen constructs release pro-inflammatory cytokines during *in vitro* culture.

## Discussion

Chronic non-healing skin wounds pose a significant health problem and are associated with extreme patient discomfort, amputations, and high mortality rates (Ellis et al., 2018). Unfortunately, the current primary treatment options for chronic skin wounds are limited to symptomatic treatments. The primary reason for poor healing outcomes of chronic wounds is the lack of adequate vascularity (Sheng et al., 2011; Liao et al., 2013; Bianchi et al., 2014). Microfat isolated from lipoaspirate is a cell-enriched fat graft consisting of ASCs and EPCs preserved in their native niche (Alharbi et al., 2013; Kokai et al., 2014; Ho et al., 2017). Injection or topical application of microfat onto a non-healing chronic wound has the potential to enhance recruitment of inflammatory cells, promote cell migration and growth, and augment neovascularization (Tremolada et al., 2016). Currently, several microfat processing devices that are capable of isolation and micronization (fragmentation) of adipose tissue are available for clinical use. One such system, Lipogems, allows for micronization of adipose tissue and preservation of cells within their native niche (Bianchi et al., 2014; Tremolada et al., 2016). However, the Lipogems system uses extensive mechanical forces with numerous washing steps during micronization which may lead to unavoidable loss of extracellular matrix (ECM) as well as reduced cellular content and viability. Several other devices use mechanical filtration to process fat which also may result in damage of ECM and integrity of fat graft clusters (Bianchi et al., 2013, 2014). The MiniTC^TM^ fat isolation and micronization device used in the current study is an efficiently designed closed-loop system that preserves the innate vasculature, minimizes loss of stromal cells, and maintains superior cell viability.

Freshly prepared microfat using the MiniTC^TM^ system is a rich source of autologous cells with a cell count of around 1 × 10^6^ cells per gram and a viability of close to 90% (Table 1). The portability of the MiniTC^TM^ device makes it feasible to use directly in the operating room for processing of patient-derived lipoaspirate and preparation of autologous microfat graft for application onto non-healing chronic wounds. Further, cryopreservation of microfat was confirmed to retain cell count and viability (Table 2) indicating that repetitive lipoaspirate collection from the patient will not be necessary; and instead, excess microfat can be cryopreserved, thawed when needed, and used for subsequent autologous application of microfat graft for repeated application to the wound site. The MiniTC^TM^ processed microfat is rich in ASCs (98%, Figure 2) which may help initiate and accelerate the regeneration process during the early phase of wound healing due to antimicrobial effect and active paracrine intercellular modulation and recruitment process. The abundance of EPCs (59.3%, Figure 2) in microfat can aid in the neovascularization process to potentially improve the ischemic condition of the chronic wound microenvironment and thereby promote wound healing. Freshly harvested SVF express different surface markers compared to the ones expressed after plating of the SVF. Specifically, higher expression of CD34 + and CD31 + markers is characteristic of EPCs in freshly harvested SVF which pose high usability to restore vascularization in the ischemic microenvironment of chronic wounds.

3D bioprinting is a layer-by-layer additive manufacturing process for the fabrication of complex tissue constructs (Cornelissen et al., 2017; Drzewiecki et al., 2018; Gopinathan and Noh, 2018). Significant advantages include precise on-demand geometric placement of biological materials within the printed construct and controlled spatial distribution of materials in a clinical setting, such as in wound dressings (Pati et al., 2014, 2015). Prior work using extrusion-based 3D bioprinting with microfat alone as the bioink has shown considerable promise (Pati et al., 2016); however, use of microfat alone without a carrier biomaterial to provide support may result in constructs with inadequate mechanical properties and poor engraftment when applied on deep full-thickness chronic wounds (Ji and Guvendiren, 2017). This work represents the first application of 3D bioprinting utilizing microfat tissue with a natural biomaterial (i.e., collagen) as a base bioink. The use of collagen as a bioink for 3D printing of microfat offers numerous advantages, including biomimetic design, preservation of cellular viability, and better retention of the microfat graft at the wound site. However, collagen as a biomaterial relies on thermal crosslinking and fibrillogenesis to form stable constructs. In addition, *in situ* photochemical crosslinking of the collagen constructs post printing is essential to ensure that the print and shape fidelity of the constructs is retained (Omobono et al., 2015; Nguyen et al., 2019; Kajave et al., 2020). Therefore, it is important to ensure that microfat incorporation does not impede the thermal crosslinking or photochemical crosslinking processes. Quantitative measurements of the printed structure (i.e., line width) showed no change in the dimensions of collagen constructs upon microfat incorporation and over time indicating that the print and shape fidelity of the constructs are preserved (Figure 3). Retention of print and shape fidelity of collagen constructs over time is important for clinical use of the printed construct as stable grafts in wound healing applications. Cell metabolic activity in microfat-laden collagen constructs was maintained for up to 10 days (Figure 4), which correlates with the time frame currently employed in the clinic between subsequent bandage changes for chronic wounds (Fonder et al., 2008; Han and Ceilley, 2017). These outcomes suggest that sequential application of 3D printed microfat-laden collagen constructs with bandage changes on a weekly basis may be an effectual approach for the treatment of non-healing wounds.

*In vivo*, wound healing takes place in four distinct phases; hemostasis, inflammatory, proliferative, and maturation phases (Ellis et al., 2018). The inflammatory phase can further be divided into the acute (consisting of primary M1-polarized macrophages) and chronic (M1-polarized macrophages transitioning into M2-polarized) inflammatory phases (Martinez and Gordon, 2014; Moore et al., 2015; Krzyszczyk et al., 2018). While each of these phases are critical for the proper healing and remodeling of the wound site, non-healing chronic wounds rarely progress beyond the acute phase of inflammation (Ellis et al., 2018; Krzyszczyk et al., 2018). There are a multitude of reasons that this may occur, including chronic infection, disease processes (e.g., diabetes), limited vascularization, or even a lack of proper chemokine regulation (Gillitzer and Goebeler, 2001; Theilgaard-Mönch et al., 2004; Monneau et al., 2016). RANTES and GM-CSF are primarily responsible for initial immune system recruitment, stimulating the active recruitment of T-cells (RANTES) and macrophages (GM-CSF) to the wound site, assisting the wound response in transitioning from the hemostasis phase to the acute inflammatory phases, and from the chronic to proliferative phases (Theilgaard-Mönch et al., 2004). IL-1β, IL-6, CXCL9, and IL-8 have been shown to be extensively involved in the initial immune response to wounds, stimulating the acute inflammatory phase and activation of M1 macrophages (Russo et al., 2014; Ellis et al., 2018). IL-6, while appearing to be stimulated in the initial acute inflammatory response, has been the subject of extensive debate, as isolated incidence of IL-6 showed anti-inflammatory properties by virtue of inhibiting the expression of TNF-α and IL-1β at initial wound sites (Kwon et al., 2014; Russo et al., 2014; Monsuur et al., 2016). Further, this progresses the wound response mechanism toward macrophage transition by stimulating expression of IL-4 and thus transitioning to the chronic inflammatory phase (Fernando et al., 2014; Volpin et al., 2014; Ellis et al., 2018). Basic fibroblast growth factor (FGF2) shows a pattern consistent with the recruitment of cells that allow transition beyond the inflammatory phase and toward the proliferative phase of wound healing, stimulating angiogenesis within the wound site (Grasys et al., 2016). ELISA results from the current study revealed a temporal expression of these cytokines from microfat in *in vitro* culture with a peak at day 3 of the culture (Figure 5). This trend correlates well with the results of the Alamar blue assay that showed a steady decline in cell metabolic activity after day 4 of the culture (Figure 4) suggesting that the decrease in cytokine secretory activity may be due to loss in cell viability in microfat with the duration of the culture and probably due to the limited space for cell expansion within a floating scaffolding structure, which is not the case upon *in vivo* application. Periodic changes (e.g., weekly) of the microfat graft of the wound site akin to bandage changes currently applied in the clinic for chronic wounds can be done to allow for sustained release of cytokines at the wound site. The release of key wound healing related cytokines from 3D printed microfat constructs is highly promising and may aid in the transition through the different stages of wound healing via enhanced cellular recruitment, neovascularization, and ultimately realization of complete healing of chronic wounds.

In conclusion, results from this work have shown that MiniTC^TM^ isolated microfat is comprised of a rich population of regenerative cells that remain viable post cryopreservation. Further, it is feasible to 3D print microfat-laden collagen constructs with well-maintained print and shape fidelity, and that these 3D printed constructs remain stable in an *in vitro* culture. Cell metabolic activity in microfat-laden collagen constructs is maintained up to 10 days in culture and microfat-laden collagen constructs release proinflammatory cytokines in culture that are known to play a key role in the different stages of the wound healing process. Future studies will entail performing more in-depth *in vitro* assessment of 3D printed microfat graft functionality (e.g., neovascularization) and carrying out *in vivo* studies to demonstrate the applicability and efficacy of these grafts in wound healing applications. Overall, combination of 3D bioprinting and cryopreserved microfat can enable the generation of custom-designed autologous microfat grafts with considerable potential for use in highly efficient, non-immunogenic, and complete healing of deep chronic wounds.

## Data Availability Statement

The raw data supporting the conclusions of this article will be made available by the authors, without undue reservation.

## Ethics Statement

Ethical review and approval was not required for the study on human participants in accordance with the local legislation and institutional requirements. The patients/participants provided their written informed consent to participate in this study.

## Author Contributions

VK and NK conceived the idea and designed the experiments. NK performed the experiments for cell count and percent cell viability, and flow cytometry analyses. TS performed all the experiments using 3D printed microfat constructs. All authors jointly compiled the figures and wrote the manuscript.

## Conflict of Interest

NK is the CEO of Jointechlabs, Inc. that owns the proprietary rights for the MiniTC^TM^ microfat isolation system. The remaining authors declare that the research was conducted in the absence of any commercial or financial relationships that could be construed as a potential conflict of interest.

## Publisher’s Note

All claims expressed in this article are solely those of the authors and do not necessarily represent those of their affiliated organizations, or those of the publisher, the editors and the reviewers. Any product that may be evaluated in this article, or claim that may be made by its manufacturer, is not guaranteed or endorsed by the publisher.
